# *Candida*-induced granulocytic myeloid-derived suppressor cells are protective against polymicrobial sepsis

**DOI:** 10.1128/mbio.01446-23

**Published:** 2023-09-08

**Authors:** Shannon Esher Righi, Amanda J. Harriett, Elizabeth A. Lilly, Paul L. Fidel, Mairi C. Noverr

**Affiliations:** 1 Department of Microbiology and Immunology, Tulane University School of Medicine, New Orleans, Louisiana, USA; 2 Center of Excellence in Oral and Craniofacial Biology, Louisiana State University Health Sciences Center School of Dentistry, New Orleans, Louisiana, USA; Texas Christian University, Fort Worth, Texas, USA

**Keywords:** polymicrobial sepsis, myeloid-derived suppressor cells, trained innate immunity, *Candida*

## Abstract

Polymicrobial intra-abdominal infections (IAI) can lead to life-threatening sepsis with significant morbidity and mortality, especially when pathogenic fungi are involved. We have employed an established clinically relevant mouse model of fungal/bacterial IAI and shown that immunization with low-virulence *Candida* species, that is, *Candida dubliniensis*, can induce responses that protect against sepsis via the suppression of lethal inflammation. This protection is dependent on long-lived Gr-1^+^ polymorphonuclear leukocytes that display characteristics consistent with myeloid-derived suppressor cells (MDSCs) and trained innate immunity. Here we aimed to functionally and phenotypically characterize these protective Gr-1^+^ leukocytes. Compared to nonimmunized control mice, we observed increased levels of CD11b^+^ Gr-1^+^ cells systemically and locally in the peritoneal cavity of immunized mice. Isolated peritoneal Gr-1^+^ cells displayed hallmark MDSC phenotypes including increased T-cell suppressor activity and increased MDSC effector activity. Furthermore, we observed increased levels of the anti-inflammatory MDSC cytokine interleukin (IL)-10 in the peritoneal cavity of immunized mice and, in contrast, increased inflammatory responses when Gr-1^+^ leukocytes were depleted from immunized mice prior to challenge. Flow cytometric analysis revealed that Ly6G^+^ granulocytic MDSCs (G-MDSCs) were preferentially increased over Ly6C^+^ monocytic MDSCs (M-MDSCs) in immunized mice. Importantly, G-MDSCs, but not M-MDSCs, as well as IL-10 production, are required for full protection against lethal sepsis. From these data, we conclude that the Gr-1^+^ leukocytes that protect against polymicrobial sepsis are bona fide MDSCs and hypothesize that the mechanism of MDSC-mediated protection includes abrogation of lethal inflammation by IL-10.

**IMPORTANCE:**

Polymicrobial intra-abdominal infections are serious clinical infections that can lead to life-threatening sepsis, which is difficult to treat in part due to the complex and dynamic inflammatory responses involved. Our prior studies demonstrated that immunization with low-virulence *Candida* species can provide strong protection against lethal polymicrobial sepsis challenge in mice. This long-lived protection was found to be mediated by trained Gr-1^+^ polymorphonuclear leukocytes with features resembling myeloid-derived suppressor cells (MDSCs). Here we definitively characterize these cells as MDSCs and demonstrate that their mechanism of protection involves the abrogation of lethal inflammation, in part through the action of the anti-inflammatory cytokine interleukin (IL)-10. These studies highlight the role of MDSCs and IL-10 in controlling acute lethal inflammation and give support for the utility of trained tolerogenic immune responses in the clinical treatment of sepsis.

## INTRODUCTION

Sepsis is defined as life-threatening organ dysfunction caused by a dysregulated host response to infection (1). This disease causes significant morbidity and mortality, with recent estimates reporting more than 48 million cases globally and 11 million sepsis-related deaths, representing nearly 20% of all deaths worldwide (2). The innate immune response during sepsis is characterized by excessive pathological inflammation leading to eventual organ failure (3). Our group developed a murine model of polymicrobial intra-abdominal infection (IAI) with *Candida albicans* and *Staphylococcus aureus* that results in acute lethal sepsis (4, 5). Intraperitoneal (IP) immunization with low-virulence *Candida* species, in particular *C. dubliniensis*, but also including *C. glabrata*, *C. auris*, and the *C. albicans efg1*∆/∆ *cph1*∆/∆ double null mutant, as well as abiotic fungal cell wall components, can induce responses that protect against polymicrobial IAI and other inducers of acute lethal sepsis (6, 7). Immunized mice exhibit lower levels of proinflammatory cytokines, reduced hypothermia, and reduced sepsis scores following sepsis challenge, indicating that protection is associated with the abrogation of inflammation (8).

Interestingly, *C. dubliniensis*-induced protection is not mediated by adaptive immune cells or trained macrophages, but rather a trained innate immune response predominated by Gr-1^+^ polymorphonuclear leukocytes (6, 7). We have postulated that these protective Gr-1^+^ cells are myeloid-derived suppressor cells (MDSCs), which are a heterogeneous population of immature immunosuppressive myeloid cells that have been identified in association with a variety of pathologic conditions, including most notably cancer, but also infectious diseases, sepsis, and inflammation (9). MDSCs are characterized by the co-expression of CD11b and Gr-1 and are therefore phenotypically similar to mature leukocytes, such as macrophages and polymorphonuclear neutrophils (PMNs). At least two subsets of MDSCs have been identified, granulocytic and polymorphonuclear MDSCs (G- and PMN-MDSCs), which in mice are CD11b^+^ Ly6G^+^ Ly6C^+/lo^ and CD11b^+^ Ly6G^-^ Ly6C^hi^ monocytic MDSCs (M-MDSCs) (10, 11). In addition, MDSCs express high levels of arginase 1 and reactive nitrogen and oxygen species and secrete anti-inflammatory cytokines including interleukin (IL)-10 and transforming growth factor beta, which are the factors that mediate their immunosuppressive functions (9). In our previous work, depletion of Gr-1^+^ cells in immunized mice abrogated protection against lethal sepsis (7, 8). The goal of these studies was to characterize the Gr-1^+^ leukocytes induced in immunized mice using a combination of flow cytometry, cell suppressor assays, enzyme activity/expression analyses, and cell/cytokine depletion studies to confirm MDSC functionality and phenotype.

## RESULTS

### Putative MDSCs are increased in the bone marrow and periphery in immunized mice

**Fig 1:**
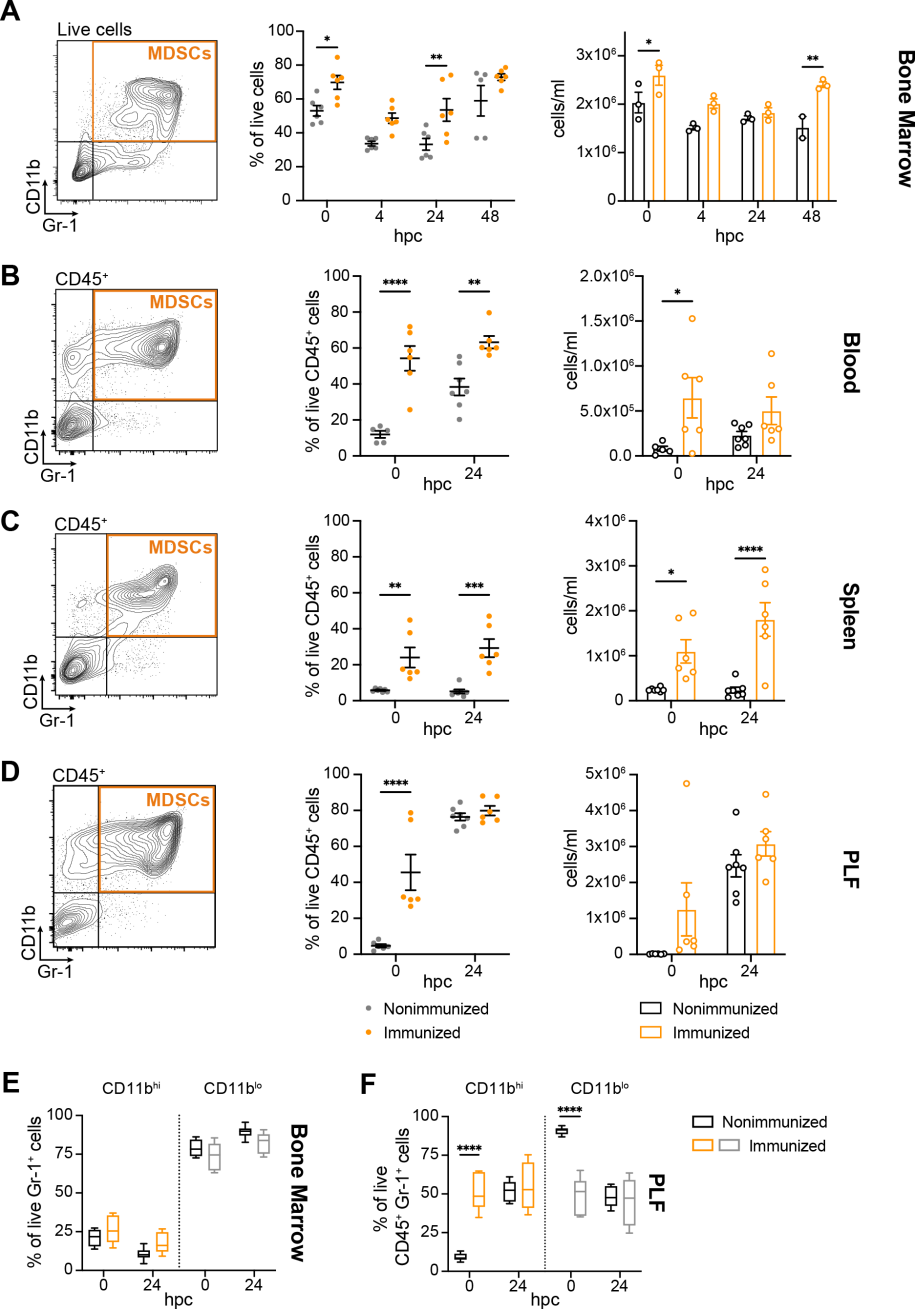
Putative MDSCs are increased following a lethal sepsis challenge. (**A**) CD11b^+^ Gr-1^+^ putative MDSCs in the bone marrow of C57BL/6 mice at 0, 4, 24, and 48 hpc (polymicrobial sepsis challenge, 7 × 10^6^–1.4 × 10^7^ CFU/mL *C*. *albicans* + 8 × 10^7^ CFU/mL *S*. *aureus*). Left, representative cell gating; middle, putative MDSCs expressed as the percentage of live cells (*n* = 2–3 mice/group/experiment; two experiments); right, putative MDSCs expressed as cells/mL (*n* = 2–3 mice/group; one experiment). (**B through D**) CD11b^+^ Gr-1^+^ putative MDSCs in the blood, spleen, and peritoneal lavage fluid (PLF) of C57BL/6 mice (*n* = 2–3 mice/group/experiment; two experiments) at 0 and 24 hpc (1.4 × 10^7^ CFU/mL *C*. *albicans* + 8 × 10^7^ CFU/mL *S*. *aureus*). Left, representative cell gating; middle, putative MDSCs expressed as the percentage of live CD45^+^ cells; right, putative MDSCs expressed as cells/mL. (**E and F**) CD11b^hi^ MDSCs and CD11b^lo^ PMNs in the (**E**) bone marrow and (**F**) PLF of C57B/6 mice (*n* = 2–3 mice/group/experiment; two experiments) at 0 and 24 hpc, expressed as the percentage of live Gr-1^+^ or live CD45^+^ Gr-1^+^ cells at each timepoint. *P < 0.05, **P < 0.01, ***P < 0.001, ****P < 0.0001, two-way ANOVA with Sidak’s multiple comparisons test. Representative full gating strategies are illustrated in Fig. S1A through C. Hpc, h post-challenge.

To extend our previous work and test the hypothesis that immunization with low-virulence *Candida* species induces an expansion of Gr-1^+^ MDSCs in the bone marrow and periphery, cell populations were analyzed by flow cytometry. The total MDSC population, defined by the presence of CD11b and Gr-1 markers, was monitored in immunized (*C. dubliniensis* IP inoculation 14 d prior) and nonimmunized mice following lethal fungal/bacterial sepsis challenge (*C. albicans + S. aureus* intra-abdominal coinfection). Results showed increases in the percentage and total number of CD11b^+^ Gr-1^+^ cells in the bone marrow of immunized mice at the time of sepsis challenge [0 h post-challenge (hpc)] and at 24 and 48 hpc, respectively, compared to nonimmunized mice (Fig. 1; Fig. S1A).

Putative MDSCs were also analyzed in the blood, spleen, and peritoneal cavity at 0 and 24 hpc. CD11b^+^ Gr-1^+^ MDSCs were significantly increased in the blood and spleens of immunized mice at both timepoints (Fig. 1B and C; Fig. S1B). In the peritoneal cavity, CD11b^+^ Gr-1^+^ and CD11b^hi^ MDSCs were significantly increased at 0 hpc in immunized mice, whereas similar levels of these cell populations were observed between groups at 24 hpc (Fig. 1D).

The level of CD11b expression was also analyzed to distinguish MDSCs from traditional PMNs, with CD11b^hi^ cells representing the MDSC fraction and CD11b^lo^ cells representing the PMN fraction (12). Results revealed a modest increase in CD11b^hi^ cells in the bone marrow of immunized mice compared to nonimmunized mice, with a modest decrease in the levels of CD11b^lo^ cells between groups (Fig. 1; Fig. S1C). No significant differences in CD11b^hi^ MDSCs or CD11b^lo^ PMNs were observed in the blood or spleens between immunized and nonimmunized mice (data not shown). By contrast, in the peritoneal cavity CD11b^hi^ MDSCs were significantly increased at 0 hpc in immunized mice, whereas similar levels of these cell populations were observed between groups at 24 hpc (Fig. 1F). CD11b^lo^ PMN levels were decreased in immunized mice at 0 hpc, but similar between groups at 24 hpc.

### Putative peritoneal Gr-1^+^ MDSCs from immunized mice inhibit T-cell proliferation

**Fig 2:**
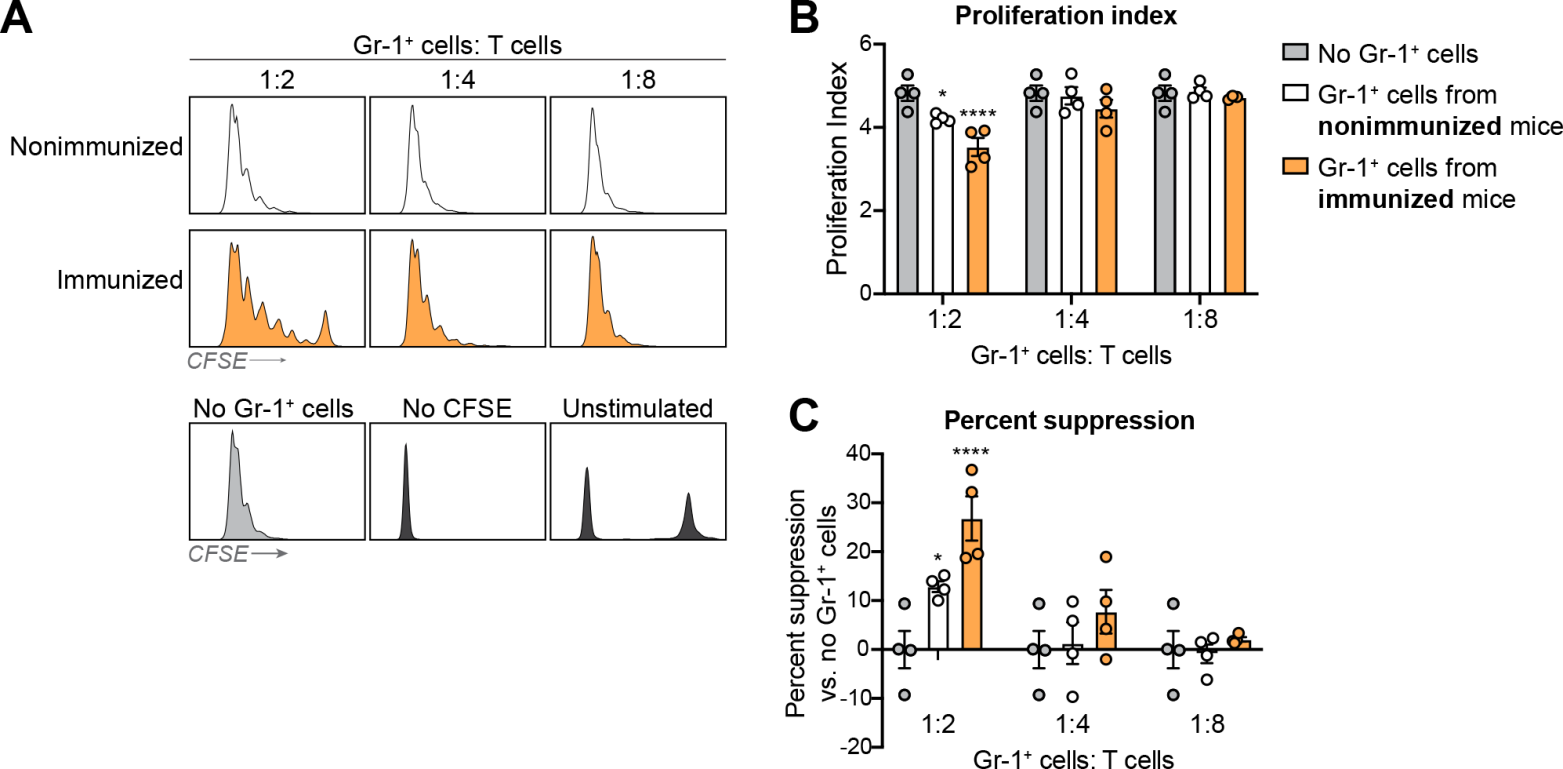
Peritoneal Gr-1^+^ cells from protected mice display enhanced immunosuppressive activity. (**A**) Proliferation of naïve, stimulated, carboxyfluorescein succinimidyl ester (CFSE)-stained, splenic CD4^+^ T cells assessed by flow cytometry after coculture for 4 d with Gr-1^+^ cells isolated from the peritoneal cavity of Swiss Webster mice (*n* = 4 mice/group, assayed in duplicate) 24 h post-abiotic septic challenge (500 mg/kg zymosan). Histograms are representative of at least four biological replicates. (**B**) Proliferation index and (**C**) percent suppression were calculated using FlowJo Proliferation Modeling. *P < 0.05, ****P < 0.0001 vs No Gr-1^+^ cells, two-way ANOVA with Dunnett’s multiple comparisons test.

To determine whether the cells present at the site of infection in the peritoneal cavity of immunized mice are functioning as bona fide MDSCs, we next measured their ability to inhibit the antigen nonspecific proliferation of CD4^+^ T cells, the gold standard assay for MDSC suppressor function (13). To avoid contamination by live microbes in this assay, Gr-1^+^ cells were isolated from the peritoneal cavity of immunized or nonimmunized mice 24 h after an abiotic septic challenge with zymosan (7). Results showed that CD4^+^ T cells cocultured with Gr-1^+^ cells from immunized mice, but not nonimmunized mice, displayed a dose-dependent reduction in proliferation (Fig. 2A). When quantified, cocultures with Gr-1^+^ cells from immunized mice showed a significant reduction in proliferation index and a significant increase in percentage of suppression when compared to cocultures with Gr-1^+^ cells from nonimmunized mice or cultures with no Gr-1^+^ cells added (Fig. 2B and C).

### Peritoneal Gr-1^+^ cells from immunized mice exhibit phenotypes consistent with MDSCs

**Fig 3:**
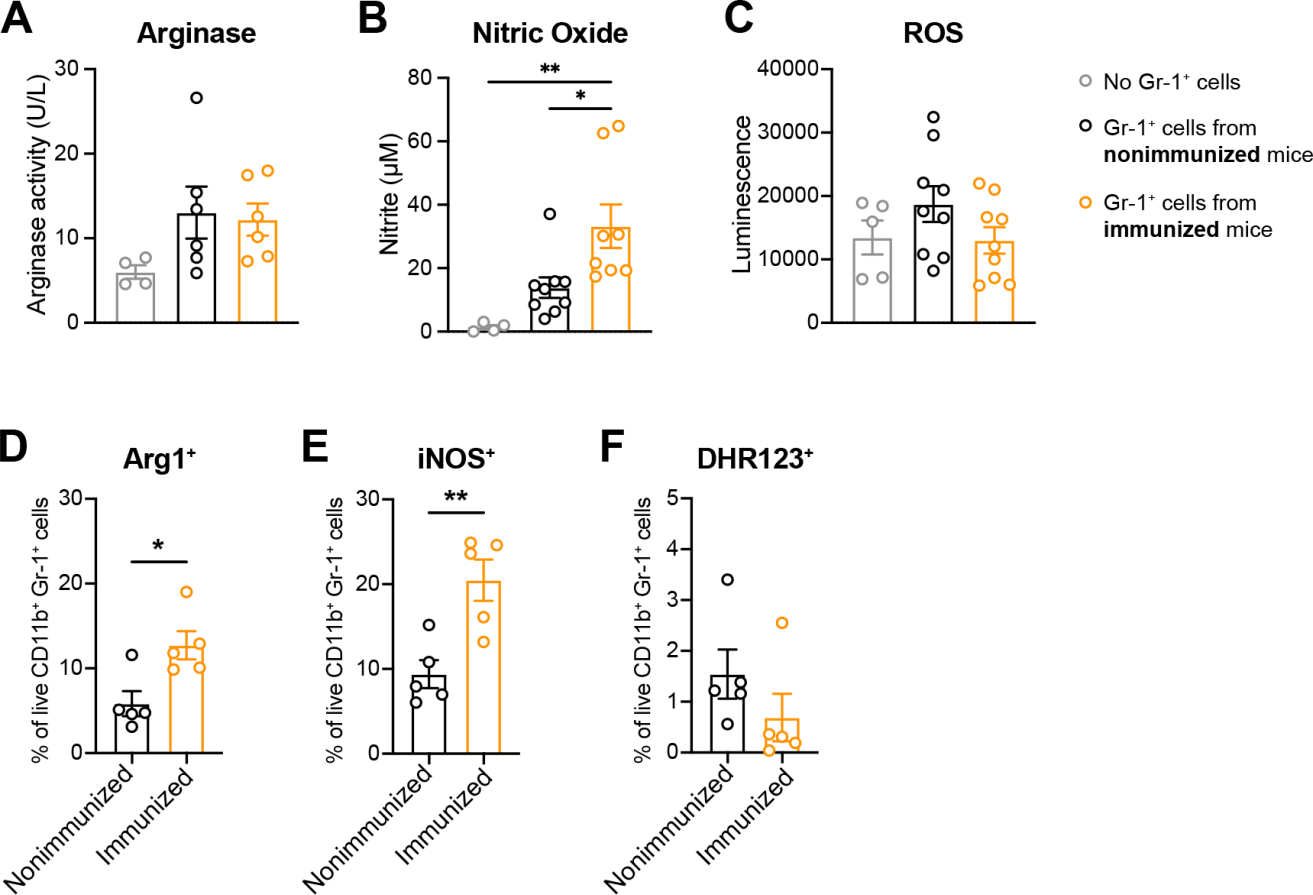
Peritoneal Gr-1^+^ cells from immunized mice exhibit MDSC-like phenotypes. (**A through C**) Production of biochemical MDSC markers by Gr-1^+^ cells isolated as above and stimulated *in vitro* for 24 h with zymosan (10 µg/mL). (**A**) Arginase activity in cell lysates (*n* = 3 mice/group/experiment; two experiments). (**B**) Nitric oxide production represented as a measurement of nitrite in cell supernatants (*n* = 3 mice/group/experiment; three experiments). (**C**) ROS generation in cell supernatants (*n* = 3 mice/group/experiment; three experiments). *P < 0.05, **P < 0.01, one-way ANOVA with Tukey’s multiple comparisons test. (**D**) Arg1, (**E**) iNOS, and (**F**) DHR123 expression assessed by flow cytometry of MDSCs (CD11b^+^ Gr-1^+^) isolated from the peritoneal cavity C57BL/6 mice (*n* = 5 mice/group; one experiment) 24 hpc (1.4 × 10^7^ CFU/mL *C*. *albicans* + 8 × 10^7^ CFU/mL *S*. *aureus*). *P < 0.05, **P < 0.01, unpaired Student’s *t*-test. Representative full gating strategies are illustrated in Fig. S2C.

To assess classical MDSC immunosuppressive effectors, peritoneal Gr-1^+^ cells isolated from immunized and nonimmunized mice 24 hpc were stimulated *in vitro* for 2 or 24 h with zymosan and assessed for arginase activity, nitric oxide (NO) production, and reactive oxygen species (ROS). Gr-1^+^ cells from immunized and nonimmunized mice had similar levels of arginase activity 24 h post-zymosan stimulation (Fig. 3A), while NO levels were significantly higher in Gr-1^+^ cells from immunized mice (Fig. 3B), and ROS production was elevated in the Gr-1^+^ cells from nonimmunized mice (Fig. 3C). At the 2 h timepoint, no significant differences in arginase or ROS were detected (Fig. S2A and B); NO production was below the limit of detection (data not shown). As a complementary method, arginase 1 (Arg1), inducible nitric oxide synthase (iNOS), and ROS expression were assessed by flow cytometry on unstimulated peritoneal cells 24 hpc. Both Arg1 and iNOS expression were significantly higher among CD11b^+^ Gr-1^+^ cells isolated from immunized mice compared to nonimmunized mice (Fig. 3; Fig. S2C), while there was no significant difference in the fluorescence of the ROS indicator dye dihydrorhodamine 123 (DHR123) between groups (Fig. 3F).

### Divergent proinflammatory and anti-inflammatory cytokine responses occur in immunized vs nonimmunized mice following sepsis challenge

**Fig 4:**
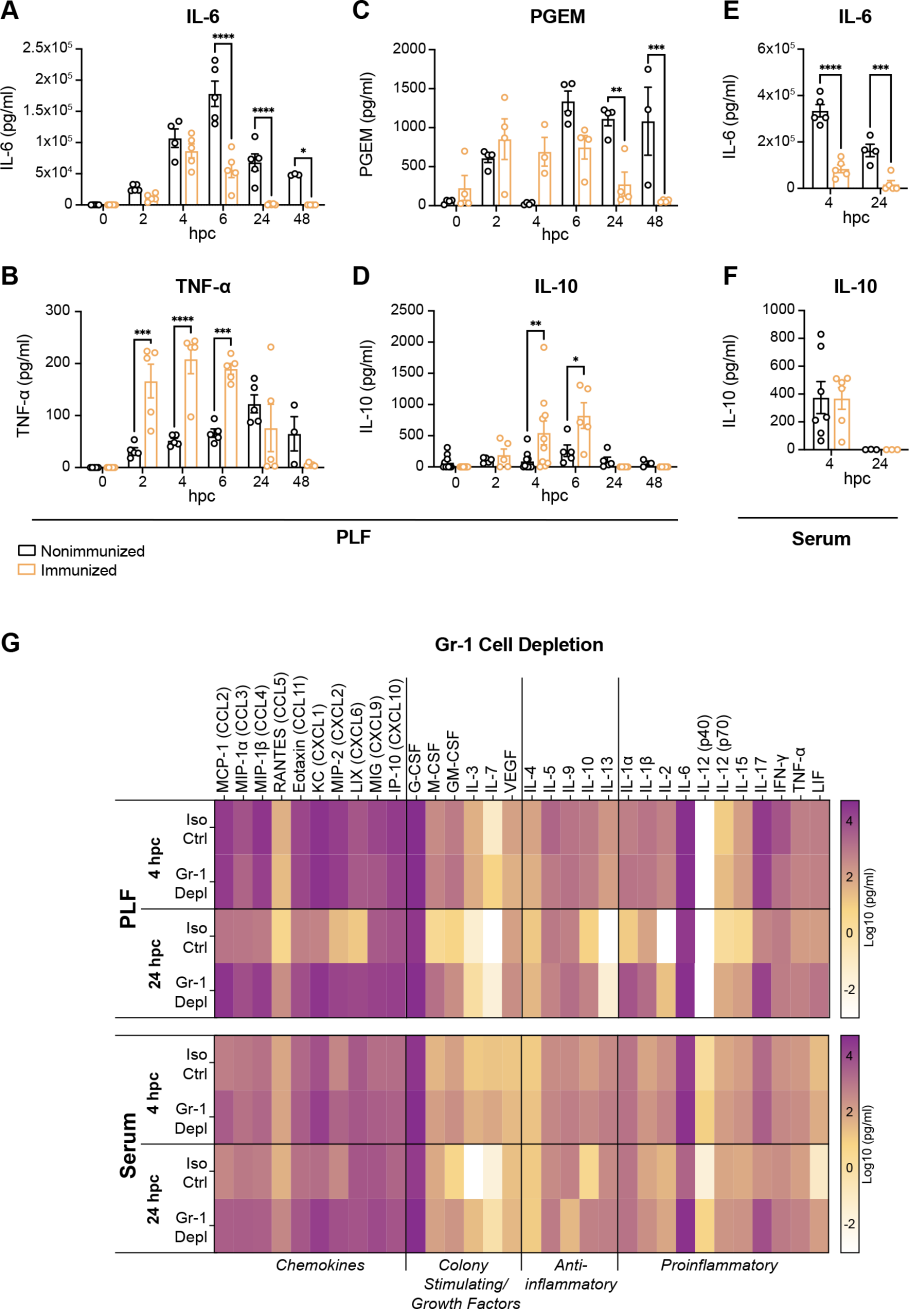
Immunized mice control the early proinflammatory response. (**A**) IL-6, (**B**) TNF-α (**C**) PGE_2_, and (**D**) IL-10 levels in the PLF, and (**E**) IL-6 and (**F**) IL-10 levels in the serum of C57BL/6 mice (*n* = 4–5 mice/group/timepoint, assayed in duplicate) at the indicated hpc (1.4 × 10^7^ CFU/mL *C*. *albicans* + 8 × 10^7^ CFU/mL *S*. *aureus*). The Prostaglandin E Metabolite (PGEM) Competitive Enzyme Immunoassay was used to measure PGE_2_ as a function of its breakdown product. *P < 0.05, **P < 0.01, ***P < 0.001, ****P < 0.0001, two-way ANOVA with Sidak’s multiple comparisons test. (**G**) Cytokine/chemokine levels in the PLF and serum of immunized Swiss Webster mice (*n* = 3 mice/group/timepoint; one experiment) following depletion of Gr-1^+^ cells. Mice were treated with 200 µg of an isotype control or anti-mouse Ly6G/Ly6C (Gr-1) antibody 48 h prior to and 2 hpc (1.75 × 10^7^ CFU/mL *C*. *albicans* + 8 × 10^7^ CFU/mL *S*. *aureus*). Data are presented as Log10 (pg/mL). Hpc, h post-challenge.

To extend our previous studies showing an association between proinflammatory cytokines and lethal sepsis (4, 5, 14), we performed kinetics of the local [peritoneal lavage fluid (PLF)] and systemic (serum) cytokine response in immunized and nonimmunized mice by ELISA. Proinflammatory IL-6 displayed rapid (within 2 hpc) and sustained elevation (out to 48 hpc) in nonimmunized mice both locally (Fig. 4A) and systemically (Fig. 4E) compared to immunized mice. Similarly, tumor necrosis factor alpha (TNF-α) levels were increased at early timepoints in immunized mice, but then declined at later timepoints compared to nonimmunized mice (Fig. 4B). PGE_2_, which is required for lethal sepsis, was also significantly higher in nonimmunized mice at the later timepoints (Fig. 4C). In contrast, levels of the anti-inflammatory cytokine IL-10 began to rise as early as 2 hpc in the peritoneal cavity of immunized mice, peaking at 6 hpc and returning to baseline by 24 hpc, while IL-10 levels were unchanged over the same time course in nonimmunized mice (Fig. 4D). IL-10 levels were not increased in the serum of immunized mice at any of the timepoints tested (Fig. 4F).

### Depletion of Gr-1^+^ MDSCs results in increased proinflammatory cytokine production in immunized mice

To further investigate the requirement for MDSCs in mediating the suppression of inflammation observed in immunized mice, Gr-1^+^ cells were depleted from immunized mice and PLF and serum were collected following lethal challenge and analyzed for cytokines/chemokines by multiplex ELISA. Compared to isotype control antibody-treated mice, immunized mice depleted of Gr-1^+^ cells had increased levels of most of the tested cytokines and chemokines locally in the PLF at 24 hpc and systemically in serum at both 4 and 24 hpc (Fig. 4G; Fig. S3). In particular, the chemokines CCL2, CCL3, CCL5, CCL11, CXCL1, and CXCL6; colony stimulating/growth factors M-CSF and vascular endothelial growth factor; and proinflammatory cytokines IL-1α, IL-1β, IL-17, and leukemia inhibitory factor (LIF) were significantly elevated locally by 24 hpc following Gr-1^+^ cell depletion, while the chemokines CCL5, CCL11, CXCL1, CXCL2, and CXCL9; colony stimulating/growth factors G-CSF and IL-7; the anti-inflammatory cytokine IL-13; and the proinflammatory cytokines IL-6, IL-15, IL-17, and TNF-α were significantly elevated systemically following Gr-1^+^ cell depletion (Fig. S3).

### G-MDSCs predominate over M-MDSCs in the bone marrow and periphery of immunized mice

**Fig 5:**
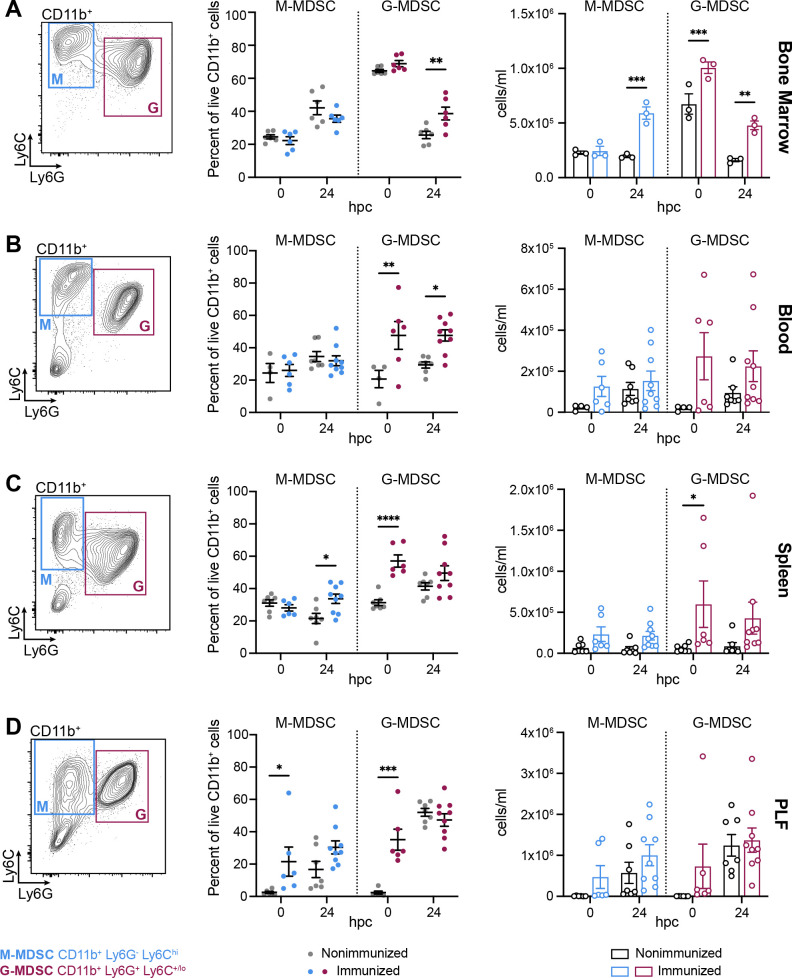
G-MDSCs are increased in immunized mice. M-MDSCs (CD11b^+^ Ly6G^-^ Ly6C^hi^) and G-MDSCs (CD11b^+^ Ly6G^+^ Ly6C^+/lo^) in the (**A**) bone marrow, (**B**) blood, (**C**) spleen, and (**D**) PLF of C57BL/6 mice (*n* = 2–3 mice/group/experiment; two experiments) at the indicated hpc (1.4 × 10^7^ CFU/mL *C*. *albicans* + 8 × 10^7^ CFU/mL *S*. *aureus*). Left, representative gates; middle, M- and G-MDSCs expressed as percentage of live CD11b^+^ cells; right, M- and G-MDSCs expressed as cells/mL. *P < 0.05, **P < 0.01, ***P < 0.001, two-way ANOVA with Sidak’s multiple comparisons test. Representative full gating strategies are illustrated in Fig. S1D. Hpc, h post-challenge.

To further characterize the Gr-1^+^ MDSCs subsets in immunized mice, G-MDSC (CD11b^+^ Ly6G^+^ Ly6C^+/lo^) and M-MDSC (CD11b^+^ Ly6G^-^ Ly6C^hi^) populations were monitored by flow cytometry following lethal fungal/bacterial sepsis challenge. In the bone marrow, there was an increased ratio of G-MDSCs prior to sepsis challenge (0 hpc) in both immunized and nonimmunized mice; however, the total number of G-MDSCs was significantly increased in the immunized mice. By 24 hpc, the percentage of G-MDSCs was significantly increased and the total number of both G- and M-MDSCs was significantly increased in the immunized mice compared to nonimmunized mice (Fig. 5; Fig. S1D). In the blood, immunized mice displayed a significant increase in the percentage of G-MDSCs at both timepoints compared to nonimmunized mice (Fig. 5B), while both the percentage and total number of G-MDSCs were significantly increased in the spleens of immunized mice (Fig. 5C). Similar to the total MDSC data (Fig. 1D), both the M- and G-MDSC subsets were significantly increased in the PLF of immunized mice at 0 hpc, while similar levels of both subsets were observed between immunized and nonimmunized mice at 24 hpc (Fig. 5D).

### G-MDSCs are required for protection against polymicrobial sepsis

**Fig 6:**
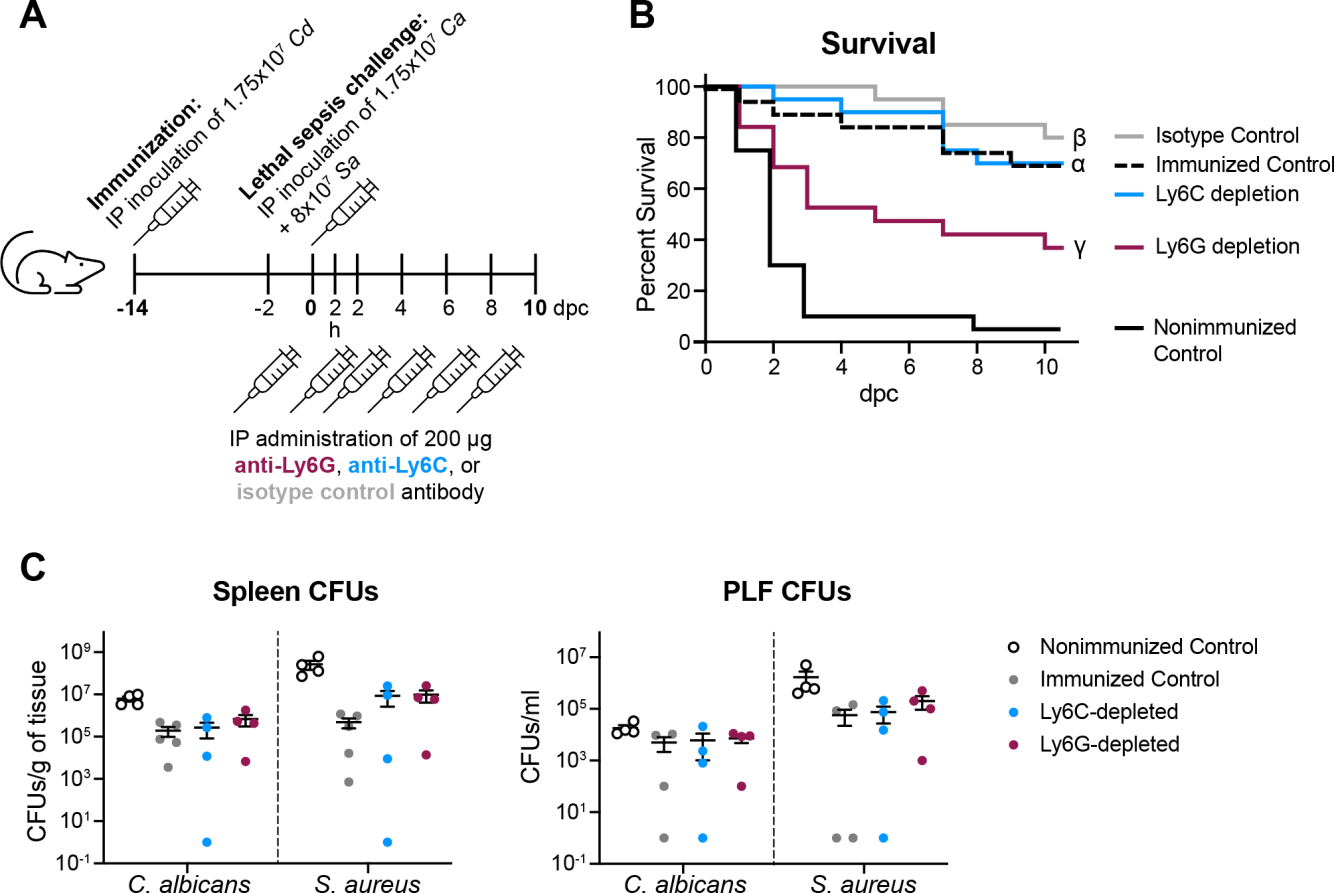
Ly6G^+^ G-MDSCs are required for protection. (**A**) Experimental design: WT Swiss Webster mice were immunized with *C. dubliniensis*, followed by lethal sepsis challenge (2.1 × 10^7^ CFU/mL *C*. *albicans* + 8 × 10^7^ CFU/mL *S*. *aureus*) 14 d later. To deplete Ly6G^+^ or Ly6C^+^ leukocytes, mice were injected IP with anti-Ly6G antibody, anti-Ly6C antibody, or isotype control antibody (200 µg) 48 h prior to and 2 h after lethal sepsis challenge, followed by every 2 d thereafter for the duration of the study. (**B**) Survival following lethal sepsis challenge (*n* = 9–10 mice/group/experiment; two experiments). α, *P* < 0.001 vs Nonimmunized control (dashed black line vs solid black line); β, ns vs Ly6C depletion (gray line vs blue line); γ, *P* = 0.009 vs Isotype control (pink line vs gray line); Log-rank test with Bonferroni correction. (**C and D**) Microbial burden in the (**C**) spleen and (**D**) PLF 2 dpc (*n* = 4–5 mice/group; one experiment). Dpc, d post-challenge.

To test the hypothesis that the expanded G-MDSC population is required for protection against lethal sepsis, immunized mice were depleted of G-MDSCs with anti-Ly6G antibodies or M-MDSCs with anti-Ly6C antibodies just prior to and during lethal challenge (Fig. 6A). Compared to immunized, isotype control antibody-treated mice, immunized mice depleted of Ly6G^+^ cells displayed rapid, early mortality (*P* = 0.009) similar to the nonimmunized septic control mice (Fig. 6B). By contrast, immunized mice depleted of Ly6C^+^ cells displayed a delayed mortality that was not significantly different from the immunized, isotype control antibody-treated mice (*P* = 1.758; Fig. 6B). In agreement with previous data demonstrating that mortality in nonimmunized mice is due to inflammation as opposed to uncontrolled microbial burden (4, 6), we found that there were no significant differences in the fungal or bacterial CFUs in the spleen or PLF of immunized control mice compared to immunized Ly6C- or Ly6G-depleted mice (Fig. 6C and D).

### *C. dubliniensis-*mediated protection against polymicrobial sepsis requires IL-10

**Fig 7:**
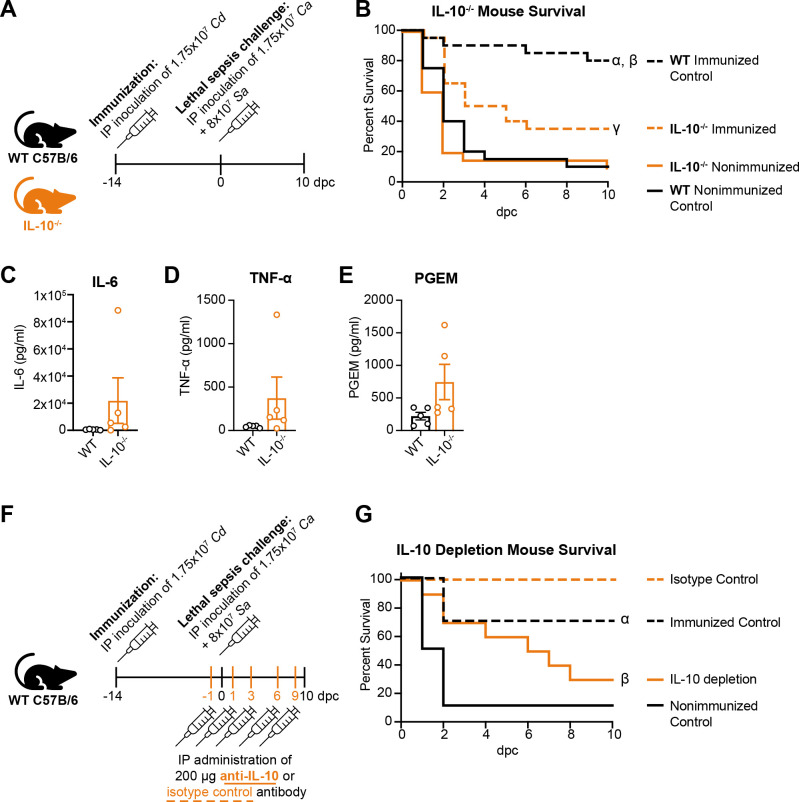
IL-10 is required for protection. (**A**) Experimental design: WT or IL-10^-/-^ C57BL/6 mice (*n* = 10 mice/group/experiment; two experiments) were immunized with *C. dubliniensis*, followed by lethal sepsis challenge (1.75 × 10^7^ CFU/mL *C*. *albicans* + 8 × 10^7^ CFU/mL *S*. *aureus*) 14 d later. (**B**) Survival following lethal sepsis challenge. α, *P* < 0.0001 vs WT nonimmunized control (dashed black line vs solid black line); β, *P* = 0.014 vs IL-10^-/-^ Immunized (dashed black line vs dashed orange line); γ, *P* = 0.025 vs IL-10^-/-^ nonimmunized (dashed orange line vs solid orange line); log-rank test with Bonferroni correction. (**C**) IL-6 (**D**) TNF-α, and (**E**) PGE_2_ levels in the PLF of immunized WT and IL-10^-/-^ mice 1 dpc (*n* = 5 mice/group; one experiment). (**E**) WT C57BL/6 mice (*n* = 10 mice/group; one experiment) were immunized with *C. dubliniensis*, followed by lethal sepsis challenge (1.75 × 10^7^ CFU/mL *C*. *albicans* + 8×10^7^ CFU/mL *S*. *aureus*) 14 d later. To deplete IL-10 during the lethal sepsis challenge phase, mice were injected IP with anti-mouse IL-10 or isotype control antibody (200 µg) on days −1, 1, 3, 6, and 9 relative to lethal sepsis challenge. (**F**) Survival following lethal sepsis challenge. α, *P* = 0.005 vs nonimmunized control (dashed black line vs solid black line); β, *P* = 0.002 vs isotype control (solid orange line vs dashed orange line); log-rank test with Bonferroni correction. Dpc, d post-challenge.

Based on our data showing an increase in IL-10 concomitant with restrained proinflammatory cytokine levels in immunized mice (Fig. 4D), we hypothesized that IL-10 production would be a requirement for MDSC-mediated protection against sepsis. To test this, survival was assessed in both *Il10*-deficient and anti-IL-10 antibody-treated immunized mice. *C. dubliniensis* immunized *Il10*-deficient mice had a significantly higher mortality following sepsis challenge compared to immunized wild-type (WT) mice (*P* = 0.0136) and nonimmunized *II10*-deficient mice (*P* = 0.025; Fig. 7A and B). The high mortality in the immunized *Il10*-deficient mice was concomitant with increased IL-6, TNF-α, and PGE_2_ in the PLF at 24 hpc compared to immunized WT mice (Fig. 7C through E). To differentiate between a requirement for IL-10 in the development of MDSCs following immunization vs a role in activated MDSC effector function during challenge, immunized mice were administered anti-IL-10 antibody only during the lethal sepsis challenge (Fig. 7F). Compared to immunized, isotype control antibody-treated mice, immunized mice depleted of IL-10 during lethal sepsis challenge had significantly higher mortality (*P* = 0.002), and similar mortality levels to nonimmunized control mice (Fig. 7G).

## DISCUSSION

MDSCs are a heterogeneous population of immature, immunosuppressive myeloid cells. Their development occurs through a two-step process beginning with expansion of the immature myeloid cell population in the bone marrow, followed by activation of these cells in the periphery and initiation of suppressive effector function (15, 16). Our previous work demonstrated that following IP immunization, *C. dubliniensis* and other low-virulence fungal species traffic to the bone marrow (17), where they can directly interact with MDSC precursors. Our data herein support the concept that *C. dubliniensis* induces both the expansion of MDSCs in the bone marrow and the trafficking to peripheral sites prior to the inflammatory insult following lethal sepsis challenge. In cancer models, the migration of MDSCs to tumor sites is driven by the chemokine receptor CXCR2 in response to CXCL1/CXCL2 (18, 19). Whether this same paradigm is true in sepsis models has yet to be determined, and the specific signals that stimulate the egress of these cells from the bone marrow to peripheral sites in our model will require further investigation.

MDSCs accumulate during sepsis clinically; however, they are generally considered to be detrimental to long-term outcomes, primarily because of their role in augmenting the immunosuppressive phase (20). By contrast, several murine models have demonstrated that accumulation of MDSCs may be beneficial, predominantly in acute or rapidly lethal sepsis models (21 – 27). Considering that our polymicrobial IAI model results in a rapid inflammatory response resembling the acute proinflammatory phase of sepsis, our data support the beneficial nature of MDSCs during this phase of sepsis. In addition, MDSCs isolated during early or late stage sepsis display different functions; early stage MDSCs were more proinflammatory and detrimental to sepsis outcomes, while MDSCs isolated during the late hypoinflammatory phase were more immature and anti-inflammatory, and were protective against sepsis when adoptively transferred during the acute phase (24, 25). The CD11b^+^ Gr-1^+^ cells that rapidly appear in the peritoneal cavity of nonimmunized mice might represent these detrimental, early-stage MDSCs, while the expanded population induced by prior *C. dubliniensis* immunization may represent the beneficial, late stage, anti-inflammatory MDSCs.

Whether this expanded MDSC population is already activated prior to inflammatory insult remains to be determined. Importantly, our functional assays (i.e., T-cell proliferation and enzyme activity/expression assays) were carried out on cells collected after lethal challenge, which was necessary because nonimmunized mice have very few cells present in the peritoneal cavity at baseline. However, we rarely observed further increases in peritoneal cells in immunized mice following lethal sepsis challenge, suggesting that the cells present are either already activated or are those that become activated and immunosuppressive. While the cells that infiltrate the peritoneal cavity of nonimmunized mice after challenge phenotypically resemble those in the immunized mice, our T-cell proliferation and enzyme activity data demonstrate they are a functionally distinct population. It is also notable that the anti-Gr-1 antibody used is not specific to MDSCs and can react with a variety of other cell types; however, the T-cell suppression phenotype and the enhanced inflammation seen when these cells were depleted from immunized mice strongly suggest that the cells targeted by this antibody are immunosuppressive MDSCs. Future work will aim to understand how the cells present in immunized mice become activated to exert their protective function.

Two subsets of MDSCs have been described in mice, granulocytic/polymorphonuclear G/PMN-MDSCs and monocytic M-MDSCs. While both subsets express arginase 1, M-MDSCs preferentially produce NO, while G-MDSCs produce higher levels of ROS (9 – 11, 28). G-MDSCs are more prevalent in tumor-bearing mice, while the ratio of each subset in humans depends on the type of cancer (29). Subset frequency in sepsis is less well defined with many clinical studies reporting conflicting results (20). This may be due to differences in the type of sepsis studied, as it has been shown that M-MDSCs predominate in gram-negative sepsis and G-MDSCs are generally higher in gram-positive sepsis (30, 31). *In vitro* studies showed a preferential induction of G-MDSCs by various *Candida* species including *C. dubliniensis* (32). In this study, G-MDSCs were increased on a systemic level in immunized mice; however, both M- and G-MDSCs were increased locally in the peritoneal cavity. Furthermore, the total peritoneal Gr-1^+^ cell population isolated from immunized mice produced higher levels of NO and reduced ROS, suggestive of an M-MDSC phenotype. Further work will be required to determine which subset is responsible for the observed *in vitro* phenotypes. However, *in vivo* antibody depletion of Ly6G^+^ leukocytes had a greater impact on *C. dubliniensis* immunization-mediated protection compared to depletion of Ly6C^+^ cells. This is in agreement with our prior studies showing that Ly6G^+^ G-MDSCs are required for protection mediated by the nonlethal *C. albicans efg1*∆/∆ *cph1*∆/∆ mutant (8). Considering the heterogeneity of Ly6C expression and the fact that G-MDSCs also express a low level of Ly6C, it is surprising that antibody depletion of Ly6C^+^ cells did not have a more significant impact on protection. Future studies will aim to further interrogate the MDSC subsets present at the site of inflammation, including assessing emerging cell surface markers and the effector phenotypes of peritoneal cells at the subpopulation level. Blocking arginase, NO, or ROS expression using small molecule inhibitors or conditional mouse knockout strains will also be important in determining the roles of these effectors in our *in vivo* polymicrobial IAI model.

IL-10 is a potent immunoregulatory and anti-inflammatory cytokine produced by a variety of immune cells, including MDSCs. In addition, IL-10 can act in a positive feedback loop with MDSCs, playing an important role in their accumulation and activation (33). Here we demonstrated that immunized mice have higher levels of IL-10 in the peritoneal cavity very early after lethal sepsis challenge compared to nonimmunized mice. This increase correlated with a decrease in hallmark proinflammatory effectors previously identified for lethal polymicrobial IAI (4, 5, 14). Interestingly, we did not observe elevated IL-10 levels in the serum of immunized mice, despite significantly decreased IL-6 levels. This indicates that (1) IL-10 production is restricted to the localized site of inflammation, and (2) this local production is sufficient to reduce systemic proinflammatory responses. We also observed an increase in the levels of hallmark proinflammatory effectors in the PLF of immunized *Il10*-deficient mice, supporting a role for IL-10 in suppressing local inflammation. We hypothesize that depletion of these proinflammatory effectors in the *Il10*-deficient background could reverse this phenotype, and future studies are planned to investigate this.

Several strategies were used to interrogate the role of IL-10 in our *in vivo* model. It is important to note that *Il10*-deficient mice lack IL-10 during both immunization and the lethal sepsis challenge. Considering the dual role that IL-10 may play in MDSC development and function, an antibody depletion approach was used to abrogate this cytokine strictly during the sepsis challenge, thereby removing any effect that lack of IL-10 might have on MDSC development. Strikingly, we observed almost identical survival between our *Il10*-deficient mice and IL-10 depletion experiments, highlighting the role of this cytokine during lethal sepsis. Whether IL-10 is produced by MDSCs directly, or if it acts indirectly on MDSCs or other cells involved in the sepsis response remains to be determined. For example, it has been reported that bone marrow stromal cells also contribute to attenuation of sepsis via production of IL-10 (34). Future studies utilizing IL-10 reporter and/or IL-10-receptor-deficient mice will aim to address these possibilities.

Finally, a major open question is how MDSCs are being trained by *C. dubliniensis*. In contrast to traditional trained immunity inducers, particularly Bacillus Calmette–Guérin vaccine and fungal β-glucan that are associated with trained monocytes/macrophages and enhanced proinflammatory responses (35, 36), protection in our model is mediated by Gr-1^+^ MDSCs and dampened inflammation (6). We have termed this form of trained immunity “trained tolerogenic immunity,” highlighting the beneficial anti-inflammatory nature of this response (37). Like MDSC development, myeloid cell training has been shown to originate in the bone marrow (38 – 40), and we have reported that protection against lethal sepsis correlates with sustained *Candida* colonization levels in the bone marrow (17). Therefore, it is plausible that the hematopoietic compartment is being trained by infiltrating *Candida* to produce an expanded MDSC population that is poised to respond to inflammatory insult. An alternative possibility is that multiple innate immune cell populations (i.e., monocytes/macrophages and MDSCs) are expanded by trained immunity inducers, and the cells that respond depend on the inflammatory insult rather than the inducer. Ongoing work is focused on interrogating the interaction and signaling between hematopoietic cells and *C. dubliniensis* that leads to the expansion/development of immunosuppressive MDSCs.

Overall, these studies complement our body of work investigating protection against sepsis by characterizing the Gr-1^+^ cells responsible for protection as bona fide MDSCs. This is highlighted by their expansion systemically and locally at the site of inflammation following protective *C. dubliniensis* immunization and suppressive effector expression/activity. Furthermore, the absence of Gr-1^+^ cells results in higher levels of inflammatory cytokines, highlighting their role as immunosuppressive cells. Finally, we show that the G-MDSC subset predominates systemically following immunization, and this subset and anti-inflammatory IL-10 are required for MDSC-mediated protection against lethal sepsis.

## MATERIALS AND METHODS

### Strains and growth conditions

The WT *C. dubliniensis* strain (Wü284) was kindly provided by Gary Moran (Trinity College, Dublin, Ireland). The WT *C. albicans* strain (DAY185) was a gift from Aaron Mitchell (University of Georgia, Athens, GA). The methicillin-resistant *S. aureus* strain (NRS383) was obtained from the Network on Antimicrobial Resistance in *S. aureus* (NARSA) data bank. Fungal strains were maintained on yeast peptone dextrose (YPD) agar and cultured in YPD broth, and bacterial strains were maintained on Trypticase soy agar and cultured in Trypticase soy broth. Standardized inocula were prepared in sterile phosphate-buffered saline (PBS) as previously described (8).

### Animal experiments

WT C57BL/6 (strain #000664) and B6.129P2-*Il10^tm1Cgn^*/J (*Il10*-deficient, strain #002251) mice (female, 5–7 weeks of age) were purchased from Jackson Laboratories. WT Swiss Webster mice (female, 5–7 weeks of age) were purchased from Charles Rivers Laboratories. Animals were housed and handled according to institutionally recommended guidelines. In some experiments, *Il10*-deficient mice were co-housed with their WT counterparts to eliminate any effect of their unique microbiome. All experiments involving animals were approved by the Tulane University Institutional Animal Care and Use Committee.

Immunized mice were administered live *C. dubliniensis* (1.75 × 10^7^ CFUs/mouse) by IP inoculation 14 d prior to sepsis challenge, as described previously (6). The animal model of polymicrobial IAI with *C. albicans* (1.4–2.1 × 10^7^ CFUs/mouse) and *S. aureus* (8 × 10^7^ CFUs/mouse) was carried out as previously described by our laboratory (4 – 6). In some cases, IP-administered zymosan A from *S. cerevisiae* (500 mg/kg in sterile saline; Sigma) was used as an abiotic inducer of lethal sepsis (7).

To deplete Gr-1^+^ leukocytes, mice were injected IP with either 200 µg anti-mouse Ly6G/Ly6C (Gr-1) antibody (clone RB6-8C5; BE0075, Bio X Cell) or rat IgG2b isotype control antibody (clone LTF-5; BE0090, Bio X Cell) in 200 µL of sterile pH 7 dilution buffer (IP0070, Bio X Cell). To individually deplete Ly6G^+^ or Ly6C^+^ leukocytes, mice were injected IP with either 200 µg anti-mouse Ly6G antibody (clone 1A8; BE0075-1, Bio X Cell) or anti-mouse Ly6C antibody (clone Monts 1; BE0203, Bio X Cell) in 200 µL of sterile pH 7 dilution buffer; 200 µg rat IgG2a isotype control antibody (clone 2A3; BE0089, Bio X Cell) in 200 µL of sterile pH 6.5 dilution buffer (IP0065, Bio X Cell) was injected IP as a control for both the Ly6G and Ly6C depletion groups. Gr-1, Ly6G, and Ly6C depletion antibodies were given 48 h prior to and 2 hpc, and then given every 2 d for the duration of the study, as previously described by our laboratory (8). Microbial burden in the spleen and PLF was assessed at 2 dpc as described previously by our laboratory (6, 8).

To deplete IL-10, mice were injected IP with either 200 µg anti-mouse IL-10 antibody (clone JES5-2A5; BE0049, Bio X Cell) or rat IgG1 isotype control antibody (clone HRPN; BE0088, Bio X Cell) in 200 µL sterile pH 7 dilution buffer. Antibody was given on days −1, 1, 3, 6, and 9 relative to lethal challenge.

### Cell isolations

Bone marrow cells (BMCs) were isolated by gently crushing mouse femurs and tibias using a mortar and pestle as previously described (41). Red blood cells (RBCs) were lysed in 1× RBC Lysis Buffer (eBioscience) for 5 min at room temperature (RT). BMCs were cryopreserved in 90% heat-inactivated fetal bovine serum (HI-FBS)/10% endotoxin-free dimethyl sulfoxide at a concentration of 0.5–2 × 10^7^ cells/mL and thawed for use as previously described (42). Splenocytes were isolated by gently mashing spleens through a 40-µm cell strainer. The strainer was rinsed with 5–10 mL PBS, cells were centrifuged at 800 × *g* for 10 min, and RBCs were lysed for 3 min at RT. Cells were obtained from the peripheral blood as previously described (43), with some modifications. Briefly, a ~100 µL sample was taken from the tail vein or via cardiac puncture and mixed with 50 µL PBS/100 mM ethylenediaminetetraacetic acid (EDTA). RBCs were lysed for 5 min on ice, inverting halfway. To isolate cells from the peritoneal cavity, peritoneal flushes were carried out as previously described (14). Cells were centrifuged at 1,500 rpm for 8 min and RBCs were lysed for 5 min at RT. Splenocytes, peripheral blood cells, and peritoneal lavage cells were resuspended in fluorescence-activated cell sorting (FACS) buffer (PBS with 2% HI-FBS and 5 mM EDTA) and stained immediately for flow cytometry.

### Flow cytometry

Single-cell suspensions (1–5 × 10^6^) were plated in round-bottom, 96-well plates and incubated with Fixable Viability Dye for 30 min. The cells were washed with FACS buffer and incubated with Fc block for 10 min, followed by cell surface staining for 30 min. Following staining, cells were washed 2× with FACS buffer and fixed with 2% paraformaldehyde for 15 min. For intracellular staining, fixed cells were permeabilized 3× with Intracellular Staining Perm/Wash buffer (Biolegend), stained for 30 min, and then washed 2× with Perm/Wash buffer. All staining steps were carried out on ice, in the dark, and stained cells were resuspended in FACS buffer for flow analysis. Antibody information and dilutions used are detailed in Table 1. Unstained cells and UltraComp eBeads Compensation Beads (Invitrogen) stained with individual fluorophores were used to calculate compensation. Fluorescence minus one controls were included as gating controls. Cells were collected on a BD LSRFortessa Flow Cytometer (BD Biosciences) and the data were analyzed using FlowJo (Tree Star) Software.

**TABLE 1:** Flow cytometry antibodies used in this study

Antibody	Fluorophore	Dilution	Clone	Vendor
Viability dye	eF506	1:1,000	-	eBioscience
Viability dye	eF780	1:1,000	-	eBioscience
CD16/CD32 (Fc block)	-	1:100	2.4G2	BD
CD45	PE-Cy7	1:100	30-F11	BD
CD11b	Alexa Fluor 488	1:100	M1/70	BD
CD11b	APC-Cy7	1:100	M1/70	BD
Ly6G/Ly6C (Gr-1)	PE	1:100	RB6-8C5	BD
Ly6G	PE-Cy7	1:100	1A8	BD
Ly6C	PE	1:100	AL-21	BD
CD3	PE-Cy7	1:100	145–2C11	BD
Arginase 1^*a*^	PerCP-eFluor710	1:100	A1exF5	eBioscience
iNOS^*a*^	eFluor450	1:100	CXNFT	eBioscience

^a^ Intracellular.

### MDSC isolation

Peritoneal Gr-1^+^ cells were isolated by positive selection with magnetic beads following the manufacturer’s protocol (Miltenyi), with some modifications adapted from references (44, 45). Briefly, 10^7^ cells were incubated with PE anti-mouse Ly-6G/Ly6C (Gr-1) antibody (1:10 dilution; clone RB6-8C5; eBioscience) for 15 min at 4°C, followed by incubation with anti-PE MicroBeads (1:4 dilution; Miltenyi) for 15 min at 4°C. Cells were subsequently passed over an MS column (Miltenyi), and purified Gr-1^+^ cells were eluted off the column in MACS buffer.

### T-cell proliferation assay

CD4^+^ T cells were isolated from splenocyte suspensions by negative selection with magnetic beads using the Miltenyi CD4^+^ T Cell Isolation Kit following the manufacturer’s protocol (Miltenyi). T cells were synchronized at 4°C overnight prior to use in proliferation assays. T cells were labeled for 5 min at RT with 25 mM CellTrace carboxyfluorescein succinimidyl ester (CFSE; Invitrogen) according to previously described methods (46, 47), resuspended in RPMI complete media (RPMI with 1× penicillin/streptomycin (P/S), 1× L-glutamine, 10% HI-FBS, 15 mM HEPES, 1× nonessential amino acids, 1 mM sodium pyruvate, 50 µM β-mercaptoethanol) supplemented with 50 U/mL recombinant mouse IL-12 (Biolegend) and plated in round-bottom, 96-well plates at a concentration of 0.8 × 10^4^ cells/well. Proliferation was induced by Mouse T-Activator CD3/CD28 Dynabeads (2 µL/well; Gibco). Peritoneal Gr-1^+^ cells were added to wells at ratios of 1:2, 1:4, and 1:8 (Gr-1^+^ cells: T cells). After 4–5 days of incubation at 37°C with 5% CO_2_, cells were harvested and viable, CD3-gated CD4^+^ T cells were analyzed for CFSE dilution by flow cytometry. To assess viability, cells were stained with 0.25 ug 7-AAD (Invitrogen) for 5 min prior to analysis.

### Arginase activity quantification, NO determination, and ROS production

Gr-1^+^ cells were isolated as above and cultured in RPMI medium (without phenol red; supplemented with 1× P/S, 1× L-glutamine, and 10% HI-FBS) at 37°C with 5% CO_2_. For NO and arginase activity determination, 2 × 10^5^ cells/well were plated in flat-bottom, 96-well plates. For ROS determination, 4 × 10^4^ cells/well were plated in flat-bottom, white opaque, 96-well plates. Cells were stimulated for 2–24 h with 10 µg/mL zymosan. Unstimulated cells and media-only wells were included as controls in all experiments.

NO production was measured in culture supernatants using Griess reagent according to the manufacturer’s protocol (Invitrogen). The absorbance was measured at 548 nm and the nitrite concentration was calculated based on a NaNO_2_ standard curve. The cell lysates were then harvested as described previously (48). Briefly, after washing with PBS, cells were lysed for 10–15 min at 37°C in lysis buffer (50 mM Tris-HCl pH 7.4, 150 mM NaCl, 1% Triton X-100, 5 mM EDTA, and 1× Thermo Scientific Halt protease inhibitor cocktail), and frozen at −20°C until use. Arginase activity was quantified in cell lysates using the Arginase Activity Assay Kit (Sigma) according to the manufacturer’s protocol. The absorbance was measured at 430 nm and arginase activity was calculated; one unit of arginase is the amount of enzyme that will convert 1 µmol of L-arginine to ornithine and urea per min at pH 9.5 and 37°C.

ROS production was measured in culture supernatants using the ROS Glo Kit (Promega) according to the manufacturer’s Homogeneous Assay (lytic) protocol. H_2_O_2_ substrate was added to the cells during zymosan stimulation, either at the beginning of the 2-h stimulation, or during the final 6 h of the 24-h stimulation. Relative luminescence was measured using a microplate reader. The ROS indicator dye DHR123 (Invitrogen) was used to assess ROS production in unstimulated peritoneal cells. Following cell surface staining (as described above under ‘Flow cytometry’), cells were incubated with a 1-µM DHR123 stock solution (prepared in FACS buffer) for 10 min at 37°C, followed by 2× washes in FACS buffer and fluorescence analysis by flow cytometry.

### Cytokine and prostaglandin analysis

Serum was collected via cardiac puncture following CO_2_ euthanasia. Whole blood was deposited into a BD Microtainer SST, allowed to clot for at least 30 min at RT, centrifuged at 10,000 × *g* for 90 s, and the upper serum layer was collected and frozen at −80°C until use. Following cardiac puncture, PLF was collected and centrifuged at 10,000 rpm for 10 min and the supernatants were frozen at −80°C until use. Concentrations of IL-6, TNF-α, and IL-10 were measured by single-plex ELISAs (Biolegend). The Prostaglandin E Metabolite Competitive Enzyme Immunoassay was used to measure PGE_2_ as a function of its breakdown product (Cayman Chemical). Multiplex mouse cytokine/chemokine concentrations were determined using the Luminex-based Milliplex MAP Mouse Cytokine/Chemokine Magnetic Bead Panel (MCYTMAG-70K-PX32, Millipore) according to the manufacturer’s instructions, and the data were analyzed using Bio-Plex Manager software (Bio-Rad).

### Statistics

Comparisons between two groups were assessed by an unpaired Student’s *t*-test. Comparisons between multiple groups were assessed by a two-way ANOVA with Sidak’s (when comparing multiple sets of means) or Dunnett’s (when comparing to a control mean) multiple comparisons test. Survival curves were compared using the log-rank test with Bonferroni correction. Significant differences were defined at a *P* value of < 0.05. All statistical analyses were performed using GraphPad Prism software.

## SUPPLEMENTAL MATERIAL

Supplemental Figure Legends mbio.01446-23-s0001.docx Legends for Fig. S1, S2, and S3.

Figure S1 mbio.01446-23-s0002.tif Gating strategies related to Fig. 1 and Fig. 5.

Figure S2 mbio.01446-23-s0003.tif Supplemental data related to Fig. 3.

Figure S3 mbio.01446-23-s0004.tif Supplemental data related to Fig. 5.

ASM does not own the copyrights to Supplemental Material that may be linked to, or accessed through, an article. The authors have granted ASM a non-exclusive, world-wide license to publish the Supplemental Material files. Please contact the corresponding author directly for reuse.
