# Supplementary material for: Candida-induced granulocytic myeloid-derived suppressor cells are protective against polymicrobial sepsis
Source: mBio. 2023 Sep 8;14(5):e01446-23. doi: 10.1128/mbio.01446-23 (PMC10653853; doi:10.1128/mbio.01446-23)
Supplement: Supplemental Figure Legends — Legends for Fig. S1, S2, and S3. [file mbio.01446-23-s0001.docx]

**SUPPLEMENTARY FIGURE LEGENDS**

**Fig. S1.** (**A**) Gating strategy for MDSCs in the bone marrow, related to Fig. 1A. Cells were gated in FSC-A vs/ SSC-A. Singlets were gated using FSC-H vs. FSC-A. Viable cells were gated and the MDSC population was defined as double positive for CD11b and Gr-1. (**B**) Gating strategy for MDSCs in the blood, spleen, and PLF, related to Fig 1B-D. Cells and counting beads were gated in FSC-A vs. SSC-A and subsequently singlets were gated using FSC-H vs. FSC-A. Viable, CD45^+^ cells were gated and the MDSC population was defined as double positive for CD11b and Gr-1. (**C**) Gating strategy for CD11b^hi^ MDSCs and CD11b^lo^ PMNs, related to Fig. 1E-F. Within the total MDSC population gated and defined as above, the CD11b^hi^ population was defined as cells with CD11b expression > 10^4^. (**D**) Gating strategy for M- and G-MDSCs, related to Fig. 5. Cells were gated in FSC-A vs. SSC-A and subsequently singlets were gated using FSC-H vs. FSC-A. Viable, CD11b^+^ cells were gated and within the live CD11b^+^ population, M-MDSCs were defined as Ly6G^-^ Ly6C^hi^ and G-MDSCs were defined as Ly6G^+^ Ly6C^+/lo^.

**Fig. S2.** (**A-B**) Production of biochemical MDSC markers by peritoneal Gr-1^+^ cells stimulated *in vitro* for 2 h with zymosan (10 µg/ml). (**A**) Arginase activity in cell lysates (n=3 mice/group/experiment; two experiments). (**B**) ROS generation in cell supernatants (n=3 mice/group/experiment; 2 experiments. (**C**) Gating strategy for MDSC marker expression, related to Fig. 3D-F. Cells were gated in FSC-A vs. SSC-A and subsequently singlets were gated using FSC-H vs. FSC-A. Live, CD45^+^ cells were gated and CD11b^+^ Gr-1^+^ MDSCs were gated. Within the MDSC population, the Arg1^+^, iNOS^+^, and ROS^+^ cell populations were defined based on fluorescence minus one controls.

**Fig. S3.** Cytokine/chemokine levels (pg/ml) in the PLF and serum of immunized Swiss Webster mice (n=3 mice/group/timepoint; one experiment) following Gr-1^+^ cell depletion, related to Fig. 4G. * p<0.05, ** p<0.01, *** p<0.001, **** p<0.0001, two-way ANOVA with Sidak’s multiple comparisons test.
