# Supplementary figures and images for: Candida-induced granulocytic myeloid-derived suppressor cells are protective against polymicrobial sepsis
Source: mBio. 2023 Sep 8;14(5):e01446-23. doi: 10.1128/mbio.01446-23 (PMC10653853; doi:10.1128/mbio.01446-23)

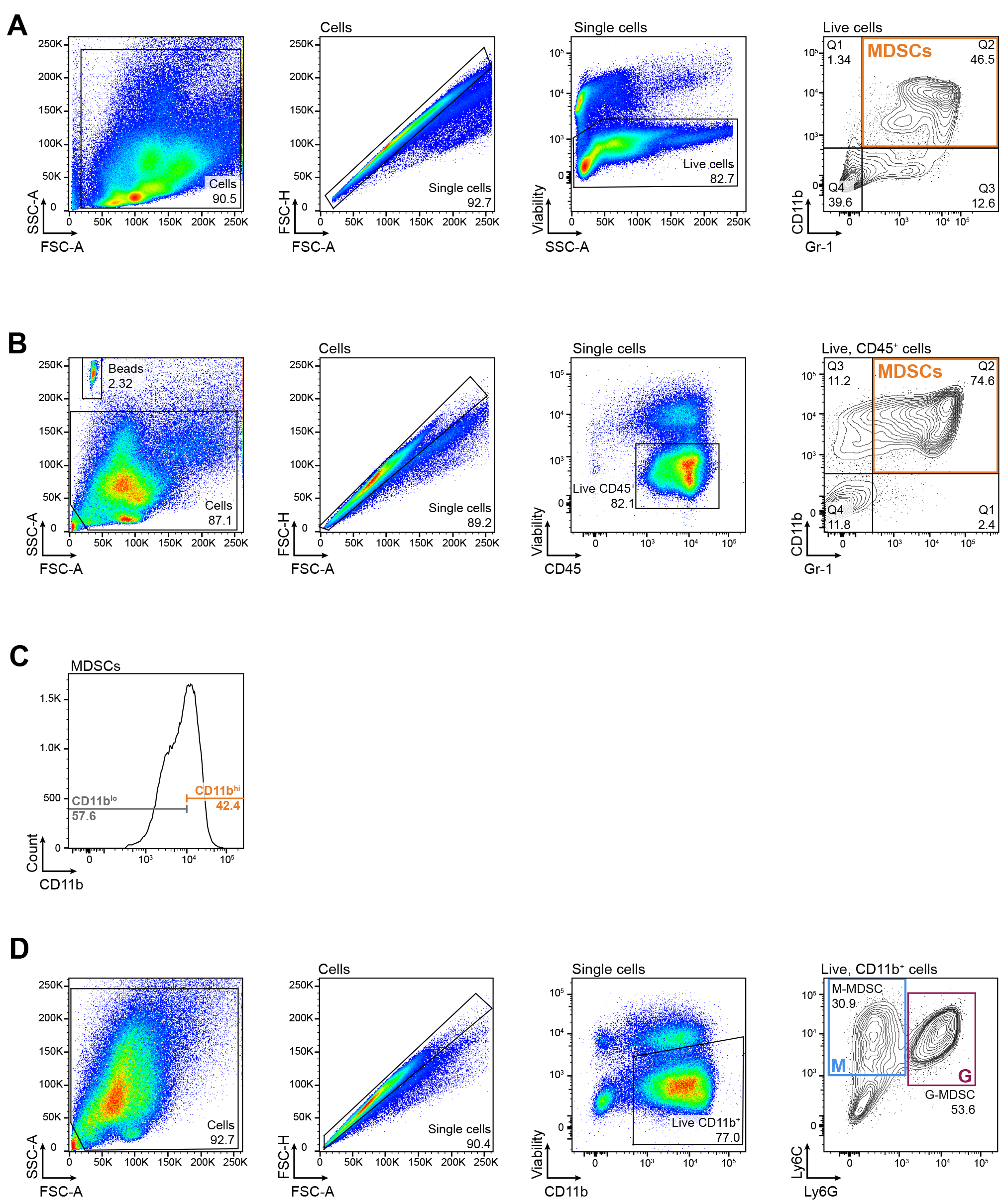

Supplement: Figure S1 — Gating strategies related to Fig. 1 and Fig. 5. [file mbio.01446-23-s0002.tif]

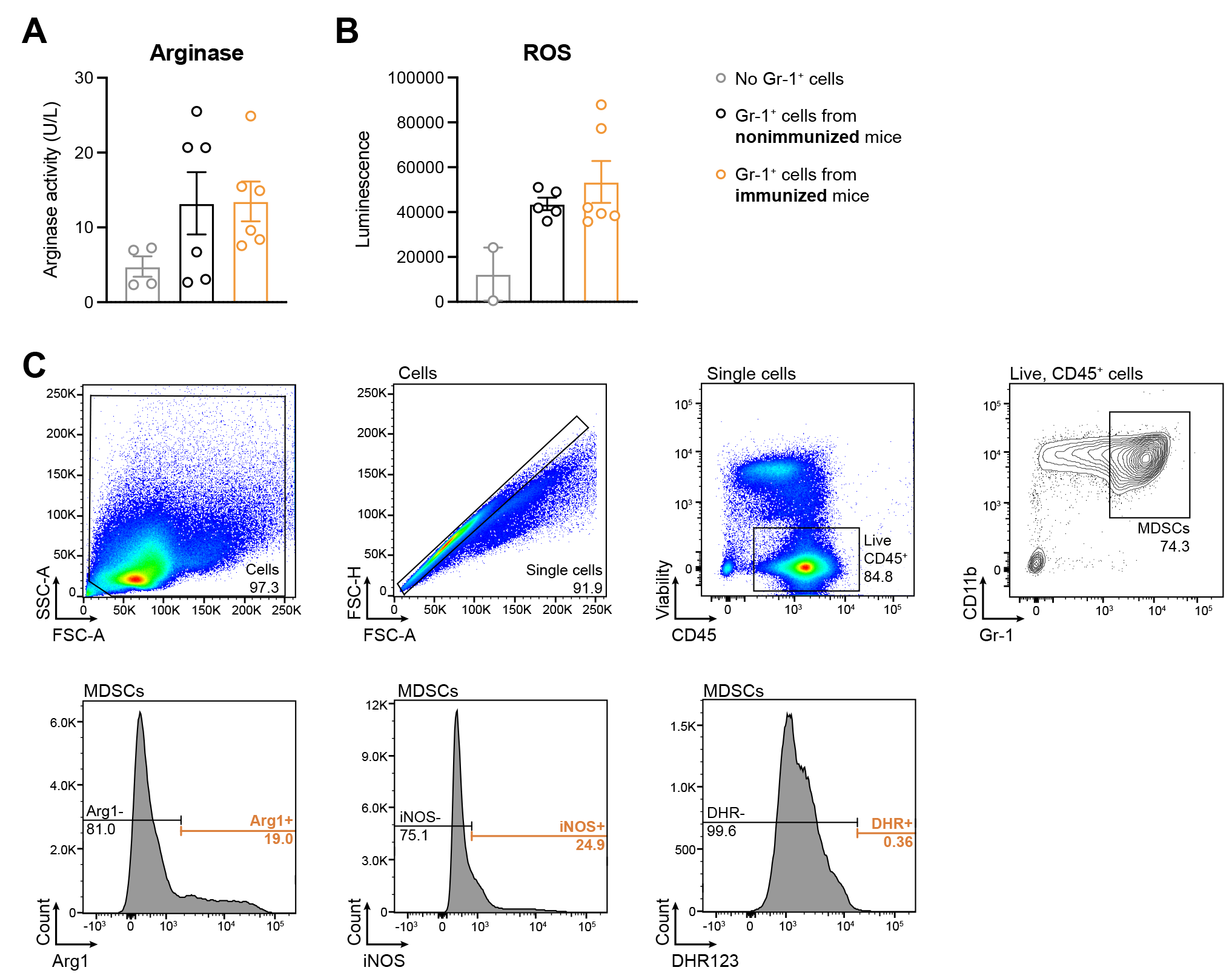

Supplement: Figure S2 — Supplemental data related to Fig. 3. [file mbio.01446-23-s0003.tif]

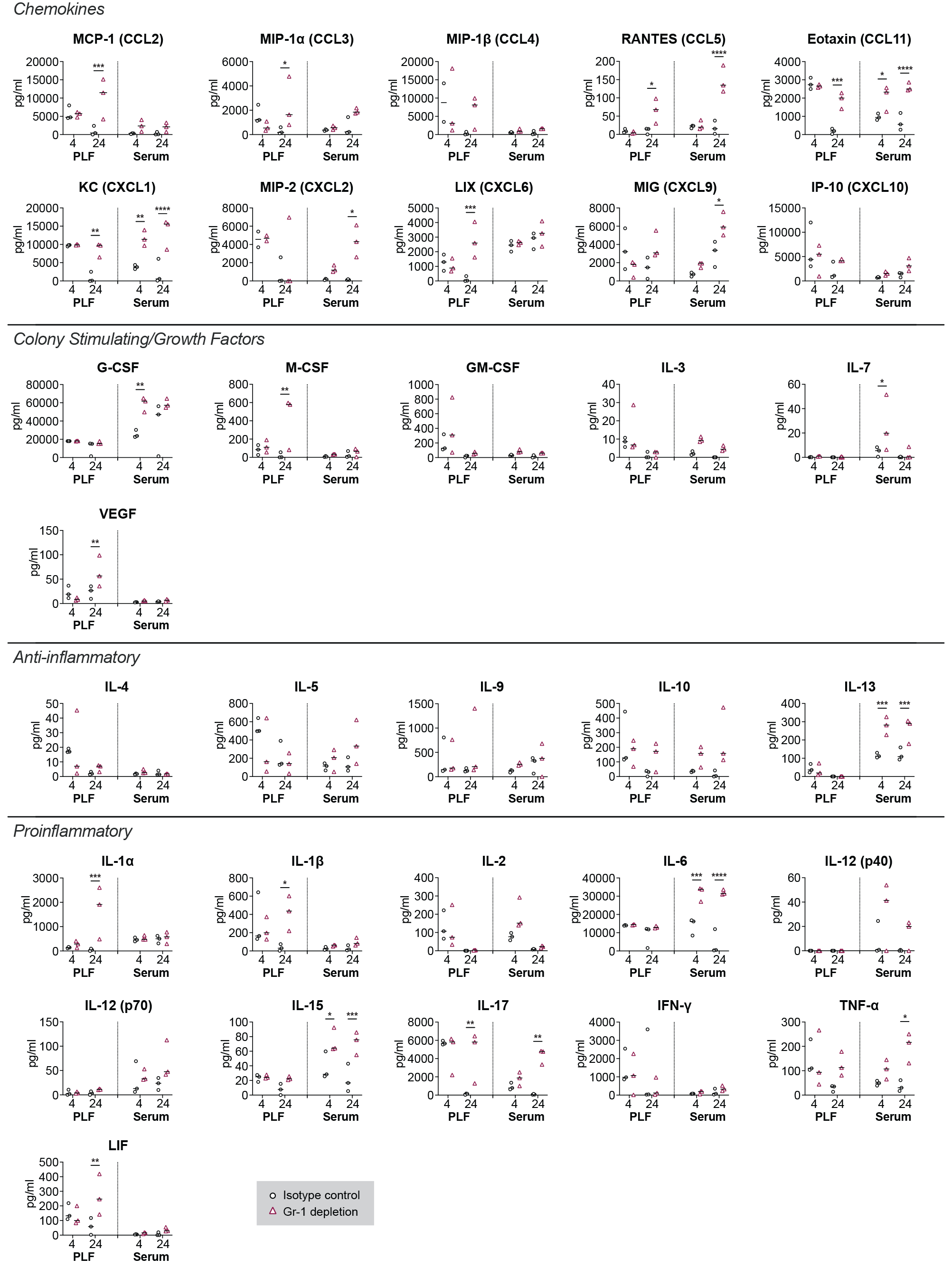

Supplement: Figure S3 — Supplemental data related to Fig. 5. [file mbio.01446-23-s0004.tif]
